# XSim: Simulation of Descendants from Ancestors with Sequence Data

**DOI:** 10.1534/g3.115.016683

**Published:** 2015-05-07

**Authors:** Hao Cheng, Dorian Garrick, Rohan Fernando

**Affiliations:** * Department of Animal Science, Iowa State University, Ames, Iowa 50011-3150; † Department of Statistics, Iowa State University, Ames, Iowa 50011-3150; ‡ Institute of Veterinary, Animal, and Biomedical Science, Massey University, Palmerston North, 4442, New Zealand

**Keywords:** genome-wide association studies, genomic prediction, pedigree, sequence data, simulation, genomic selection, shared data resource, GenPred

## Abstract

Real or imputed high-density SNP genotypes are routinely used for genomic prediction and genome-wide association studies. Many researchers are moving toward the use of actual or imputed next-generation sequence data in whole-genome analyses. Simulation studies are useful to mimic complex scenarios and test different analytical methods. We have developed the software tool XSim to efficiently simulate sequence data in descendants in arbitrary pedigrees. In this software, a strategy to drop-down origins and positions of chromosomal segments rather than every allele state is implemented to simulate sequence data and to accommodate complicated pedigree structures across multiple generations. Both C++ and Julia versions of XSim have been developed.

Analysis of real or imputed genotypes for genomic prediction and genome-wide association studies can result in findings that are difficult to validate. Simulated data have advantages in that the underlying causal mutations and simulated breeding values are available for direct validation. In general, there are two types of simulation methods: coalescent methods and forward-in-time (drop-down) methods. Compared to coalescent-based simulations, forward-in-time simulations are very flexible and allow modeling large numbers of recombination events in concert with complex life-like selection scenarios (Chadeau-Hyam *et al.* 2008; Hoggart *et al.* 2007). However, forward-in-time methods, which drop allele states down the pedigree to simulate and record genomic information for every individual in the entire population, are computationally intensive (Hoggart *et al.* 2007). Here, a strategy is described to drop-down origins and positions of chromosomal segments rather than every allele state to efficiently simulate sequence data and complicated pedigree structures across multiple generations. The software tool XSim, which incorporates our efficient strategy, has been developed to use founders characterized by real genome sequence data and complicated pedigree structures among descendants.

## Materials and Methods

### Simulation method

The basic idea of our strategy is to record the starting positions and founder origins (founder chromosome identifiers) of each chromosome segment rather than the allele state at each locus for the whole genome.

At first, entire chromosomes in founders are labeled with unique identifiers. Without considering mutations, each chromosome in each descendant individual can be represented using a pair of vectors: a vector of crossover positions and a vector of founder origins. In addition to the position and origin vectors, the allele states of the founder genomes need to be either generated from user-defined map positions and allele frequencies or obtained from real haplotypes or sequence data. As explained in the example below, during meiosis, the gamete that is formed will contain chromosomal segments from the paternal and maternal chromosomes of the parent with new segments introduced on either side of any crossover sites.

**Figure 1:**
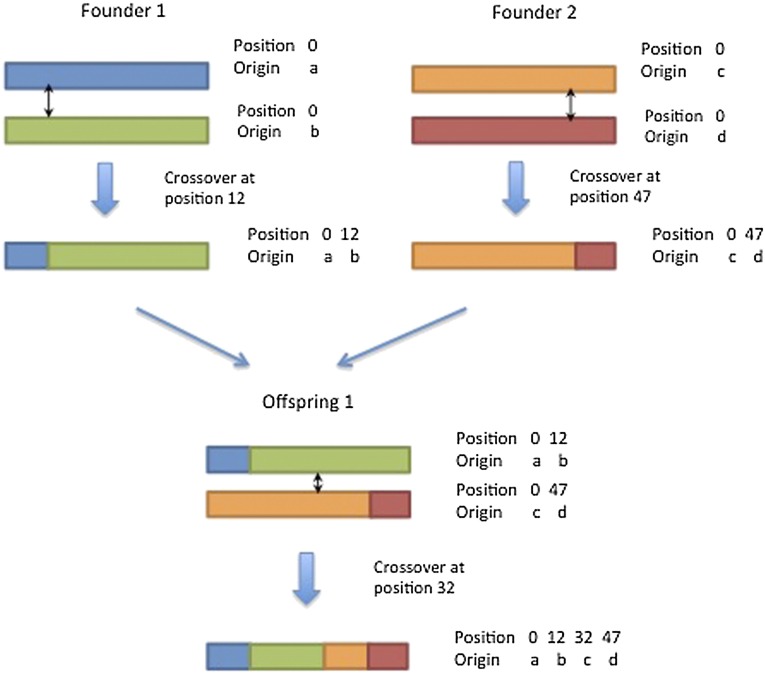
An example to illustrate the simulation strategy (crossover sites indicated by ↕).

An example to illustrate the simulation strategy is shown in Figure 1. The pairs of starting base pair position and origin vectors for founder 1 are {[0], [a]} and {[0], [b]}. During meiosis, assuming a crossover occurs at base pair position 12 (*e.g.*, 12.0 Mb), the pair of position and origin vectors for one of the resulting recombinant chromosomes is {[0, 12], [a, b]}. Similarly, pairs of position and origin vectors for founder 2 are {[0], [c]} and {[0], [d]}. Assuming a crossover occurs at base pair position 47 (*e.g.*, 47.0 Mb), the pair of position and origin vectors for one of the resulting recombinant chromosomes is {[0, 47], [c, d]}. Thus, the chromosomes of the offspring of founder 1 and founder 2 are {[0, 12], [a, b]} and {[0, 47], [c, d]}. Suppose a crossover occurs at base pair position 32 during meiosis in this offspring. Then, the pair of position and origin vectors for one of the resulting recombinant chromosomes is {[0, 12, 32, 47], [a, b, c, d]}. Then, given the positions of the crossover sites and corresponding origins of chromosomes, the entire genome of any nonfounder can be constructed to the density of the founder genomes. In the classical gene drop method, all allele states are dropped-down sequentially from founders all the way to the last generation. However, in the drop-down strategy proposed here, what are dropped-down sequentially from founders to the last generation are sparse vectors containing only founder origins and crossover positions. Thus, in the absence of mutation, computing time and memory requirement to drop-down this genomic information over generations is free of the number of loci. Once the origin and position vectors are available in the latter generations of interest, allele state information from founders can be dropped-down directly to this generation.

As one can observe from the example, in each meiosis, position and origin vectors will grow in size due to new crossover sites. When the paternal and maternal chromosome segments have the same founder origins at the crossover site, the position of the crossover site is not recorded in the resultant recombinant chromosomes. The probability of this happening is inversely related to the effective population size. Sometimes crossover events will result in reducing the length of the position and origin vectors. It can be observed that the length of these two vectors plateaus to a constant that depends on the effective population size (Goddard 2008). Thus, when the effective population size is approximately 100, for example, and the number of loci being simulated on each chromosome is more than 1000, our simulation method will be much faster than the classical gene-drop method, which sequentially simulates the passage of all allele states from founders to last generation.

Besides crossover positions and founder origins, mutations can be tracked by recording in an additional vector the positions of inherited and *de novo* mutation sites for each chromosome in each individual. The growth rate of the “mutation” vectors depends on the mutation rate and the number of loci being simulated. Unlike the position and origin vectors, the length of mutation vectors keeps growing. When the length of the mutation vectors becomes too long for efficient computation, allele states from founders can be dropped-down directly to current nonfounders. Then, these nonfounders can be relabeled and therefore treated as new founders. Now, their chromosomes will be labeled with unique identifiers, and this reduces the length of origin and position vectors to one. For these individuals, the length of mutation vectors will be reduced to zero. This strategy has been adopted in XSim.

In summary, three vectors are used to represent each chromosome in each nonfounder: the first vector to record crossover positions, the second to record origins of chromosomes, and the third to record mutation sites. This strategy is efficient because these vectors are sparse relative to the allele state vectors used in the classical gene drop approach.

### Software tool

In the C++ software tool, three hierarchical C++ classes referred to as LocusInfo, ChromosomeInfo, and GenomeInfo were defined to specify genetic characteristics at locus, chromosome and genome levels. These classes can be used to specify user-defined parameters such as allele frequencies, map positions, number of loci, chromosome lengths, numbers of chromosomes, and mutation rates. Values for these parameters can also be generated randomly.

Three C++ classes, Animal, Cohort, and Population, are defined to simulate the passage of the information on collections of individuals over generations. Complex mating structures such as cross breeding, overlapping generations, and arbitrary user-defined pedigrees are straightforward. In XSim, real haplotype data, such as from the 1000 human genomes project, can be used for founders rather than limited user-defined parameters.

XSim has also been implemented in Julia, a new dynamic programming language. The performance of Julia is often similar to that of C++. Compared to C++, software tools written in Julia are more user-friendly.

## Discussion

This article describes an efficient strategy to simulate descendants forward in time from ancestors with any density of variant information up to and including sequence data, which can be obtained for founders by sequencing or simulation. This strategy has been implemented in both C++ and Julia versions of XSim. The software tool XSim incorporating this efficient strategy has been developed to use founders characterized by any density of variant information and complicated pedigree structures among descendants.

Several forward-in-time simulation packages have been developed to simulate sequence data. Similar strategies to drop-down origins and positions of chromosomal segments have been described (Haiminen *et al.* 2013; Aberer and Stamatakis 2013; Kessner and Novembre 2014) and implemented in packages such as forqs (Kessner and Novembre 2014). These packages, however, are not developed to accommodate simulations from ancestors with real sequence data. In forqs, the growth of position and origin vectors are considered to be linear with number of generations due to recombination. However, in our simulation strategy, the length of position and origin vectors plateaus to a constant. Fregene is also an efficient forward-in-time simulation package. It is efficient when all loci in founders are homozygous. Fregene, however, does not accommodate simulations from ancestors with real sequence data (Hoggart *et al.* 2007).
